# Corrigendum: Diabetic cornea wounds produce significantly weaker electric signals that may contribute to impaired healing

**DOI:** 10.1038/srep46997

**Published:** 2018-06-27

**Authors:** Yunyun Shen, Trisha Pfluger, Fernando Ferreira, Jiebing Liang, Manuel F. Navedo, Qunli Zeng, Brian Reid, Min Zhao

Scientific Reports 6; Article number: 2652510.1038/srep26525; published online: 06 10 2016; updated 06 27 2018

The Acknowledgements section in this Article is incomplete.

“This work was supported by NIH EY019101 to M.Z. This study was supported in part by an Unrestricted Grant from Research to Prevent Blindness, Inc., and an NEI core grant. Y.S. is supported by a fellowship from the China Scholarship Council. F.F. is supported by Fundação para a Ciência e Tecnologia (FCT) grant SFRH/BD/87256/2012. M.F.N. is supported by NIH HL098200 and HL121059. We thank Dr. James Jester (UC Irvine) for the generous gift of hTCEpi cells. We are grateful to Bradley Shibata and Dr. Paul Fitzgerald, Dept. of Cell Biology and Human Anatomy, UC Davis, for help with cornea histology (NEI Core facilities Grant P30 EY012576, PI, John S Werner), and Madeline Nieves, Dept. of Pharmacology, UC Davis, for technical support. We also thank Dr. Vijay Raghunathan (Surgical and Radiological Sciences, UC Davis) for help with cell culture and imaging. We are also grateful to Dr. Rivkah Isseroff and Michelle So (Dermatology, UC Davis) for helpful discussions and initial tissue samples”.

should read:

“This work was supported by NIH EY019101 to M.Z. This study was supported in part by Air Force Office of Scientific Research under award number FA9550-16-1-0052, R21EB015737, and an Unrestricted Grant from Research to Prevent Blindness, Inc.. Y.S. is supported by a fellowship from the China Scholarship Council. F.F. is supported by Fundação para a Ciência e Tecnologia (FCT) grant SFRH/BD/87256/2012. M.F.N. is supported by NIH HL098200 and HL121059. We thank Dr. James Jester (UC Irvine) for the generous gift of hTCEpi cells. We are grateful to Bradley Shibata and Dr. Paul Fitzgerald, Dept. of Cell Biology and Human Anatomy, UC Davis, for help with cornea histology (NEI Core facilities Grant P30 EY012576, PI, John S Werner), and Madeline Nieves, Dept. of Pharmacology, UC Davis, for technical support. We also thank Dr. Vijay Raghunathan (Surgical and Radiological Sciences, UC Davis) for help with cell culture and imaging. We are also grateful to Dr. Rivkah Isseroff and Michelle So (Dermatology, UC Davis) for helpful discussions and initial tissue samples”.

